# Supplementary material for: Experimental evolution of Saccharomyces cerevisiae for caffeine tolerance alters multidrug resistance and target of rapamycin signaling pathways
Source: G3 (Bethesda). 2024 Jul 11;14(9):jkae148. doi: 10.1093/g3journal/jkae148 (PMC11373655; doi:10.1093/g3journal/jkae148)
Supplement: jkae148_Supplementary_Data [file jkae148_supplementary_data.zip › Supplemental_Figure_Legends_G3-2024-405078.docx]

**Figure S1:** Doubling times for ancestors and caffeine-evolved clones. For 0mM caffeine, all differences from ancestors are *p* ≥ 0.05 by ANOVA with Tukey’s HSD, except YMD4688, *p* = 0.0004. For 20mM and 40mM caffeine, non-significant difference from ancestor denoted by “ns;” all other clones have *p* < 0.0001 except YMD4692 at 20mM (p = 0.0003), YMD4705 at 20mM (p = 0.018), YMD4712 at 40mM (p = 0.0002), and YMD4726 at 40mM (p = 0.0001).

**Figure S2:** (A) Doubling times in 20mM and 40mM caffeine for ancestors and caffeine-evolved clones without nonsynonymous mutations. Differences are not statistically significant from ancestor by ANOVA with Tukey’s HSD. (B) Copy number inferred from read coverage of 1000bp windows. Arrows indicate alterations confirmed using IGV. (C) Doubling time of clones grouped by mutations in multidrug resistance genes; data from Fig. 2C without ancestor, none, and other groups. Difference between groups by ANOVA with Tukey’s HSD, ** p < 0.01.

**Figure S3:** (A) Correlation between doubling time of caffeine-evolved clones and ancestors in caffeine and clotrimazole. (B) Growth curves of ancestors and caffeine-evolved clones in the presence of clotrimazole to test for cross-resistance. Each curve represents one clone and is the average of 3 biological replicates. None indicates evolved clones with no mutations detected. (C) Comparison of number of evolved clones with mutations, including *PDR5* amplification, in different PDR family genes from caffeine and clotrimazole experimental evolution. (D) Growth of diploid strains with heterozygous *PDR1* mutations. (E) Growth in caffeine of clones from clotrimazole evolutions with gain-of-function mutations in *PDR1* and *PDR3*. (F) Additional replicates of lacZ reporter assay. (G) Growth in caffeine of haploid or (H) heterozygous diploid CRISPR engineered strains with synonymous *YRR1*^T696=^ mutation, with or without *YRR1*^T699P^ mutation.

**Figure S4:** (A) Relative growth of clones in presence vs. absence of 5nM rapamycin, measured by optical density after 24 hours compared to growth in caffeine. (B) Growth of strains with indicated deletions in caffeine media.

**Figure S5:** (A) Growth of haploid and (B) heterozygous diploid strains with indicated mutations in media containing caffeine. Each contains a synonymous mutation, see Table S1 for details. (C) Doubling time of diploid strains from B.
