# Supplementary figures and images for: Experimental evolution of Saccharomyces cerevisiae for caffeine tolerance alters multidrug resistance and target of rapamycin signaling pathways
Source: G3 (Bethesda). 2024 Jul 11;14(9):jkae148. doi: 10.1093/g3journal/jkae148 (PMC11373655; doi:10.1093/g3journal/jkae148)

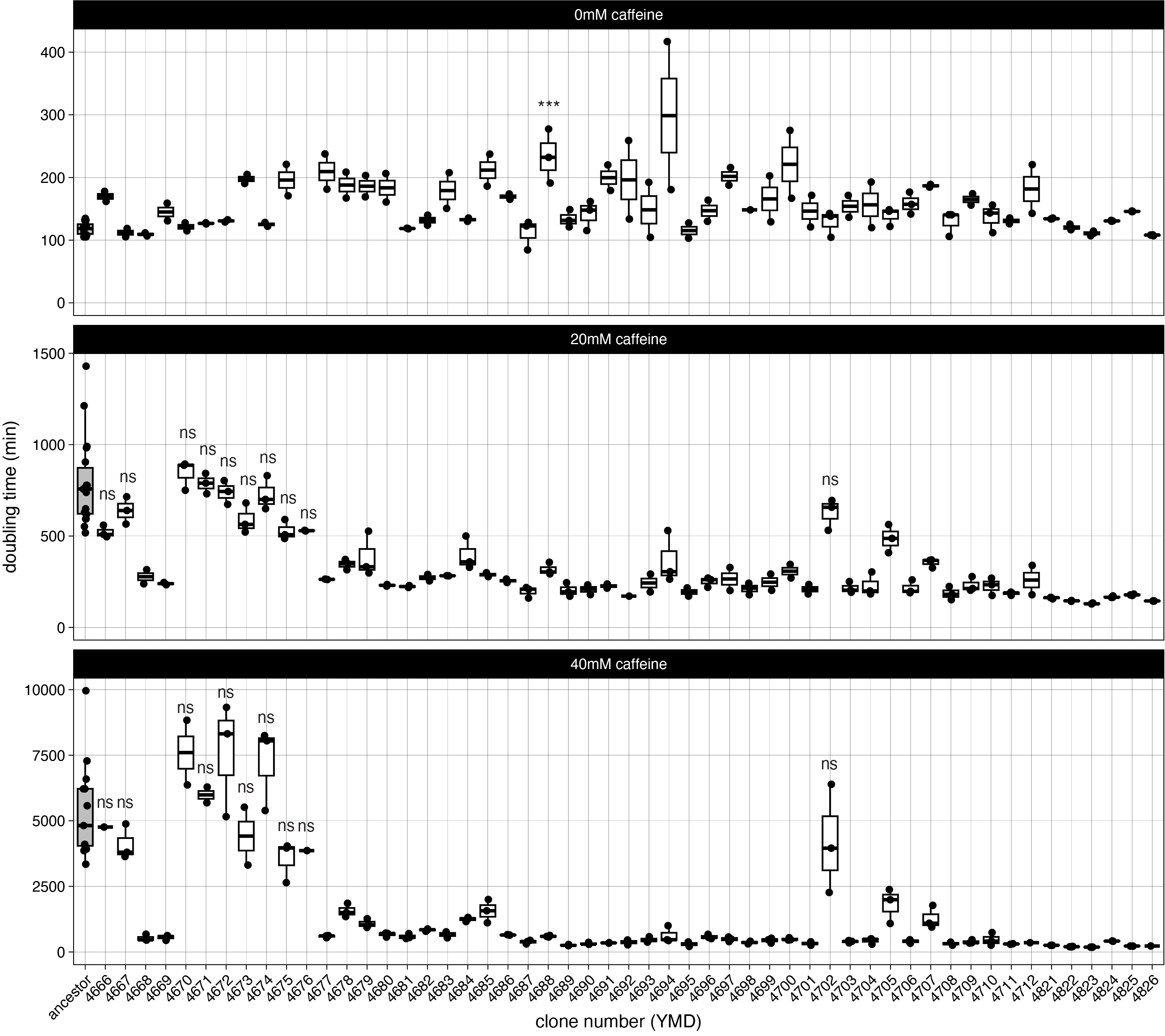

Supplement: jkae148_Supplementary_Data [file jkae148_supplementary_data.zip › Figure_S1_G3-2024-405078.png]

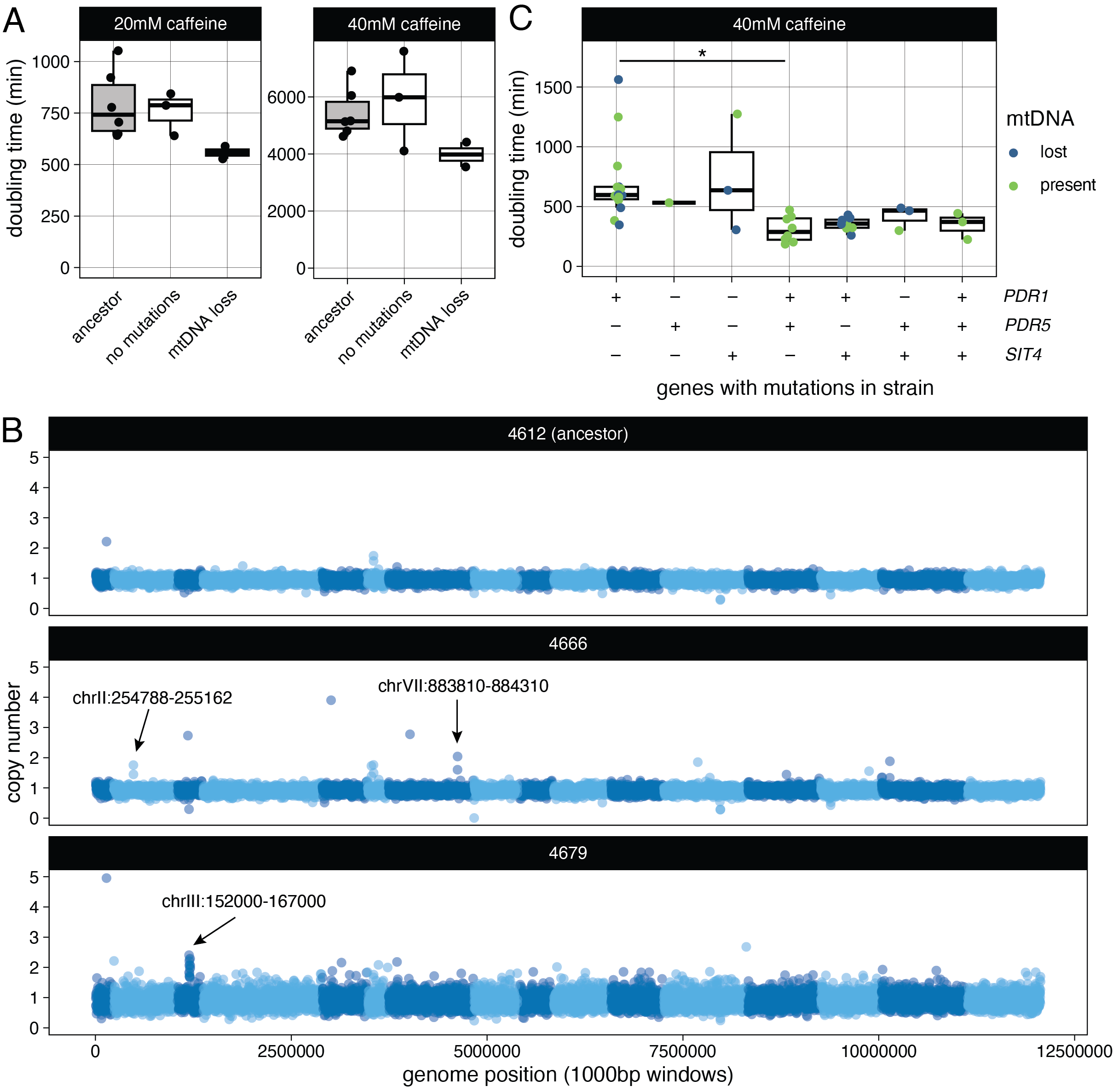

Supplement: jkae148_Supplementary_Data [file jkae148_supplementary_data.zip › Figure_S2_G3-2024-405078.png]

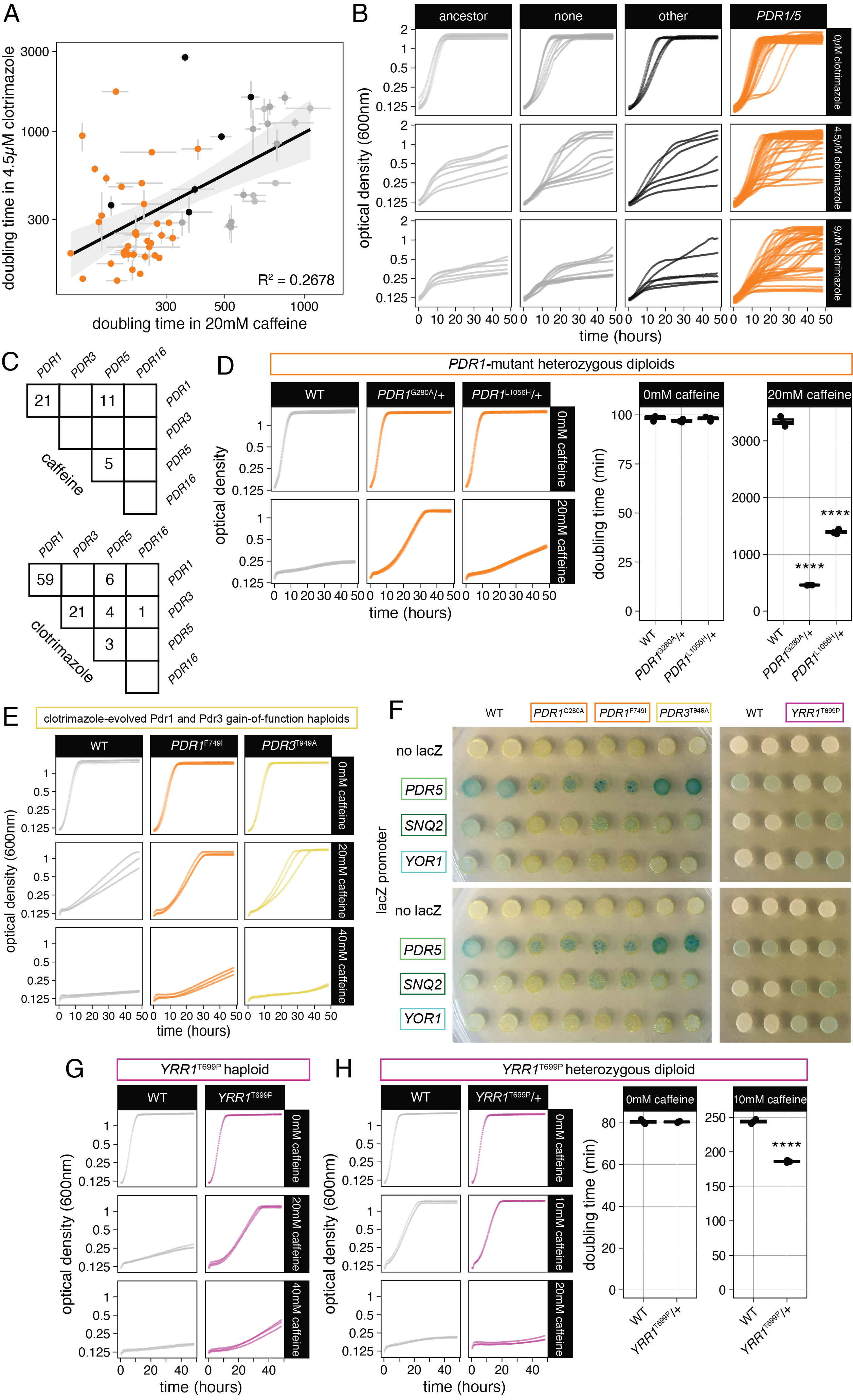

Supplement: jkae148_Supplementary_Data [file jkae148_supplementary_data.zip › Figure_S3_G3-2024-405078.png]

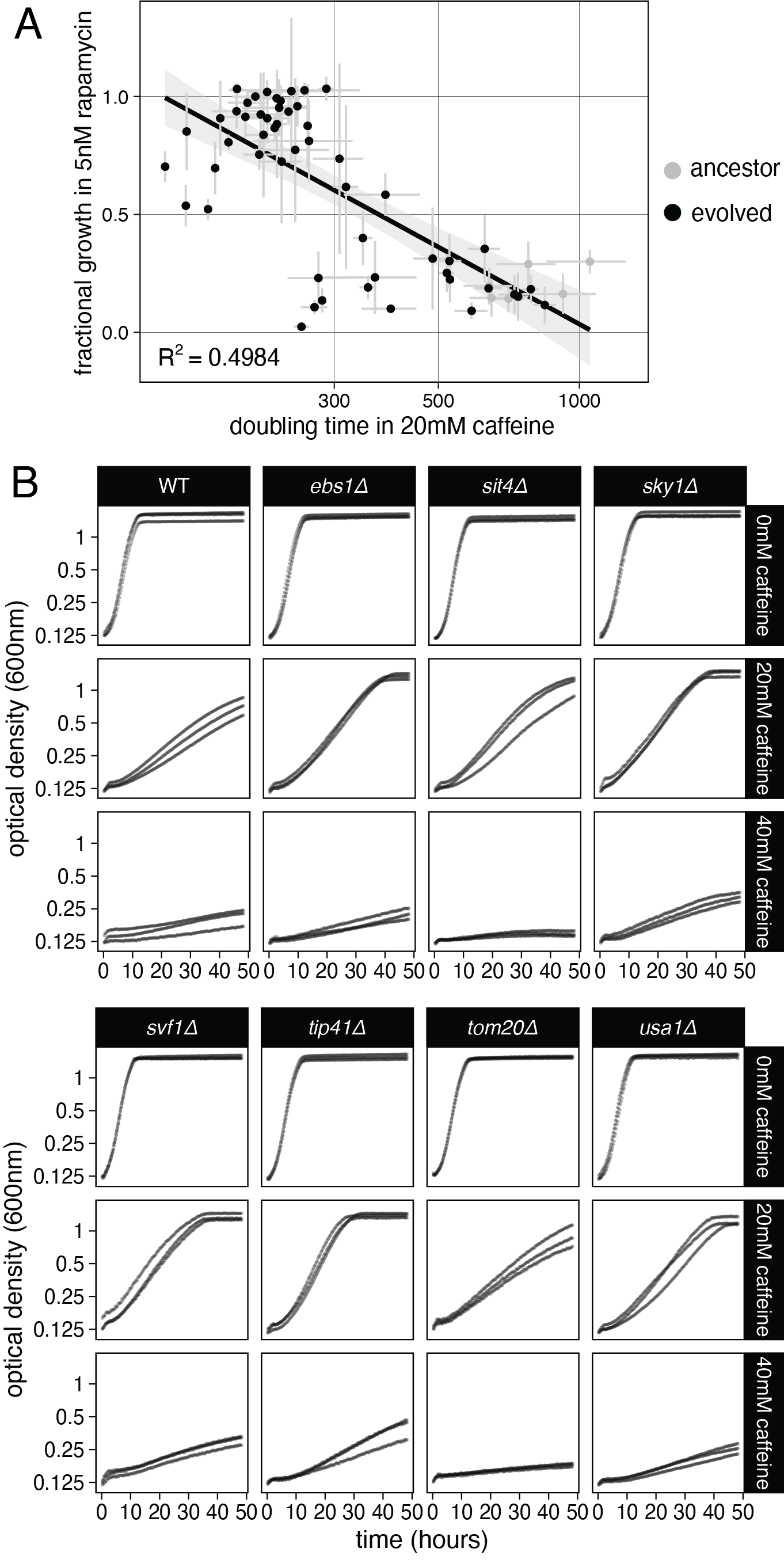

Supplement: jkae148_Supplementary_Data [file jkae148_supplementary_data.zip › Figure_S4_G3-2024-405078.png]

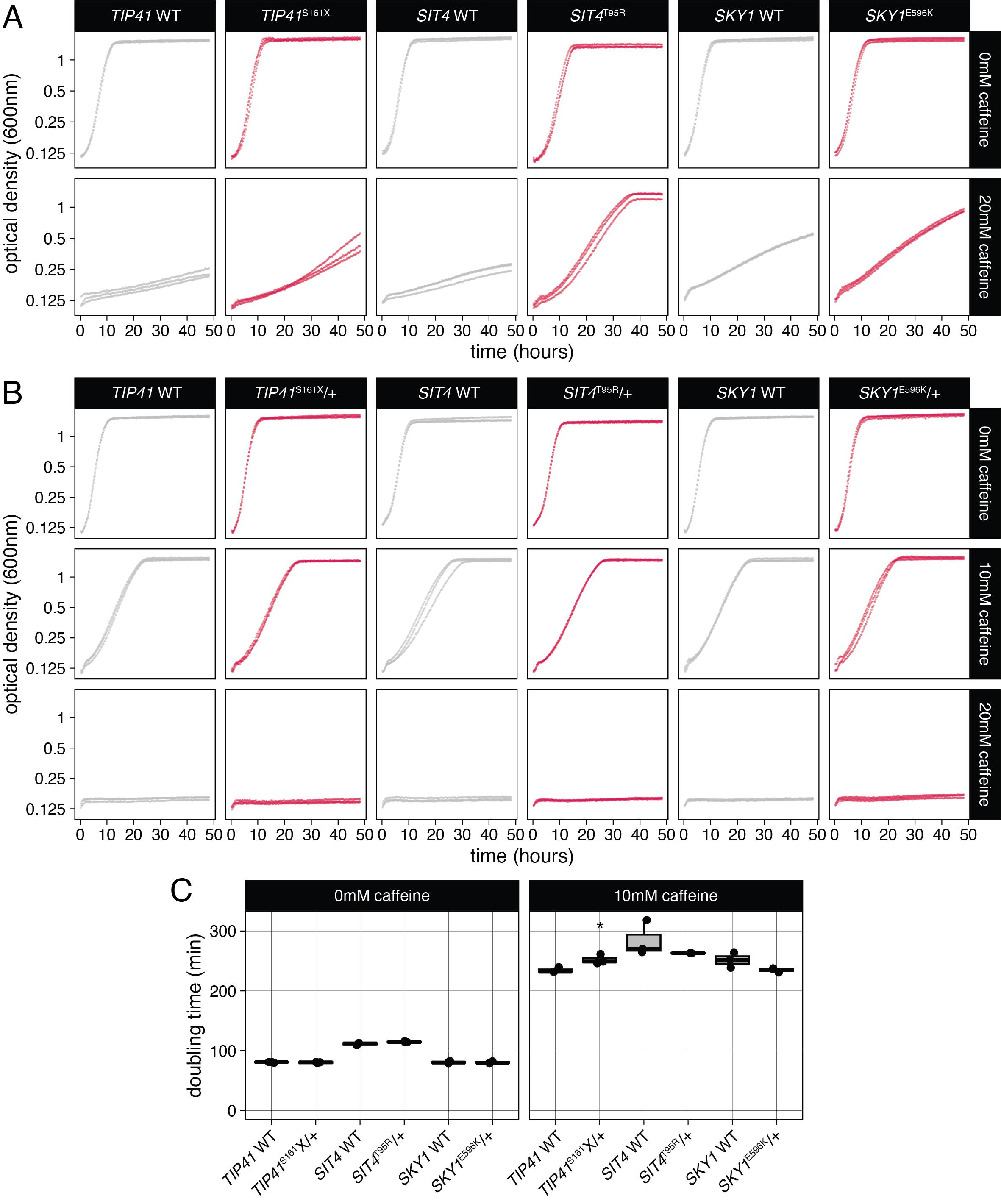

Supplement: jkae148_Supplementary_Data [file jkae148_supplementary_data.zip › Figure_S5_G3-2024-405078.png]
